# A Mixed-Methods Investigation of Medical Follow-Up in Long-Term Childhood Cancer Survivors: What Are the Reasons for Non-Attendance?

**DOI:** 10.3389/fpsyg.2022.846671

**Published:** 2022-03-14

**Authors:** Mareike Ernst, Elmar Brähler, Jörg Faber, Philipp S. Wild, Hiltrud Merzenich, Manfred E. Beutel

**Affiliations:** ^1^Department of Psychosomatic Medicine and Psychotherapy, University Medical Center of the Johannes Gutenberg-University Mainz, Mainz, Germany; ^2^Department of Pediatric Hematology, Oncology, and Hemostaseology, Center for Pediatric and Adolescent Medicine, University Medical Center of the Johannes Gutenberg-University Mainz, Mainz, Germany; ^3^Preventive Cardiology and Preventive Medicine-Department of Cardiology, University Medical Center of the Johannes Gutenberg-University Mainz, Mainz, Germany; ^4^Center for Thrombosis and Hemostasis, University Medical Center of the Johannes Gutenberg-University Mainz, Mainz, Germany; ^5^German Center for Cardiovascular Research (DZHK), Mainz, Germany; ^6^Institute for Medical Biostatistics, Epidemiology and Informatics, University Medical Center of the Johannes Gutenberg-University Mainz, Mainz, Germany

**Keywords:** cancer survivorship, childhood cancer, follow-up, health care, qualitative, mixed methods, long-term survival

## Abstract

As long-term childhood cancer survivors (CCS) are at risk for late effects, ongoing medical care is crucial to detect and treat physical illnesses as early as possible. However, previous research from around the world has shown that many adult survivors did not participate in long-term medical follow-up. This study aimed to provide insight into German survivors’ care situation, with a particular focus on barriers to follow-up care. We investigated a sample of adult CCS (*N* = 633) (age *M* = 34.92; *SD* = 5.70 years) drawn from the German Childhood Cancer Registry’s oldest cohort (> 25 years after diagnosis). Our analyses included data from a standardized medical examination, a self-report questionnaire, and in-depth interviews with a subsample (*n* = 43). Half of the participants (*n* = 314, 49.6%) reported participating in some kind of medical follow-up. In a logistic regression analysis, attendance of medical follow-up care was associated with higher age. Reasons for non-attendance were assigned to four categories: lack of information about medical follow-up and/or its purpose (*n* = 178), termination by the health care provider (*n* = 53), structural barriers (*n* = 21), and emotional-motivational aspects (*n* = 17). The interviews contributed to a better understanding of how these reported barriers played out in the care of individual survivors. Further, they revealed that some survivors currently in medical follow-up had had periods without follow-up care in the past—which were also in many cases related to a lack of information, both on the part of health care providers and CCS themselves. The results indicated that a large proportion of long-term CCS do not receive the recommended follow-up care. Further, there is a great need for more information regarding the aims of long-term medical follow-up and available offers. This is an important prerequisite for CCS to make informed decisions.

## Introduction

The growing numbers of long-term childhood cancer survivors (CCS) have brought considerations of their wellbeing in adulthood into focus (Van Dongen-Melman, 2000; Chow et al., 2020). Long-term survival rates currently range around 80% (Gatta et al., 2014), however, CCS are prone to mental (Friend et al., 2018) and physical health issues (e.g., cardiovascular and endocrine late effects): In the Childhood Cancer Survivor Study, adult CCS’ risk for severe or life-threatening conditions was more than eight times the risk of their siblings (Oeffinger et al., 2006). Risk-stratified aftercare programs have been developed and coordinated by experts around the world, e.g., based on cohort studies, in the context of PanCareSurFup/PanCareLIFE (Winther et al., 2015; Michel et al., 2019) and by The International Late Effects of Childhood Cancer Guideline Harmonization Group [IGHG] (2022). However, despite general agreement about its necessity, the implementation of long-term follow-up care into the health care system varies considerably by country. International studies, e.g., based on large samples of Swiss cancer survivors, have shown that up to 75% of long-term CCS did not receive the recommended care (Michel et al., 2011).

Several reasons have been suggested: Many CCS feel physically well and late effects may not manifest until decades later. Thus, CCS’ care needs are not apparent to medical professionals without the relevant expertise (Landier et al., 2006; Suh et al., 2014). Also, as CCS grow older, they face the challenge to transition from pediatric to adult care. This is a critical phase and care can be interrupted or terminated, especially if health care providers do not collaborate (Rosenberg-Yunger et al., 2013; Howard et al., 2018; Nandakumar et al., 2018; Daly et al., 2019). Correspondingly, studies have found that young adult CCS’ attendance of medical surveillance decreased with age (Klosky et al., 2008; Berg et al., 2016). Other characteristics that were associated with participation in follow-up in large, registry-based samples care were female gender (Oeffinger et al., 2004) and socioeconomic factors, including lower socioeconomic status (Klosky et al., 2008) and having medical insurance (in United States-based studies) (Oeffinger et al., 2004). In addition, the need to travel long distances made it harder for CCS living in large countries such as Canada, Australia, and the United States to continue care (Barakat et al., 2012; Nathan et al., 2016; Nandakumar et al., 2018; Daly et al., 2019). Motivational variables were important as well. Emotional barriers (including fear of pathological findings) could outweigh perceived benefits of medical follow-up, such as the prevention of negative consequences of the illness (Casillas et al., 2010). For example, a Canadian interview study (Rosenberg-Yunger et al., 2013) found that some survivors’ anxiety was so strong that it prevented them from attending the recommended care.

With regard to German CCS, risk-stratified guidelines for standardized long-term follow-up care were published by the German society for pediatric oncology and hematology (GPOH) (Schuster et al., 2013; currently under revision). Depending on the former patients’ condition, treatment, age, and other individual differences, they recommend medical examinations (such as an echocardiogram, ultrasonography, neuropsychological testing) and intervals for regular check-ups (Gebauer et al., 2020). Exemplary, for those with low risk for late effects, medical follow-up care could be offered by their general physician (GP) and they should also attend specialized late effects clinics every 5 years. However, there is currently no data available that informs about German CCS’ motives and barriers regarding medical follow-up care. This constitutes a research gap as it is not clear whether results from other countries are transferable to the German context, for instance due to different health care systems.

We aimed to extend previous research by investigating reasons for non-attendance of medical follow-up care in depth. To this end, we integrated quantitative questionnaire data and qualitative analyses of semi-structured interviews. Based on the available evidence, we expected that follow-up care attendance would decrease with age. Given the German context, we further expected that health care disparities [such as lack of health insurance in United States-American studies (Barakat et al., 2012)] were not the most important reasons for non-attendance. Instead, we expected emotional-motivational barriers and attitudes to play an important role.

## Materials and Methods

This work was part of the interdisciplinary studies CVSS (Cardiac and Vascular late Sequelae in long-term Survivors of Childhood Cancer, clinicaltrials.gov-nr. NCT02181049) and its add-on PSYNA (Psychosocial long-term effects, health behavior and prevention among long-term CCS). The research was carried out in accordance with the ethics standards of the institutional research committee and with the Declaration of Helsinki. It was approved by the local ethics review committee [Rhineland-Palatinate Chamber of Physicians, nr. 837.453.13 (9138-F)].

### Participants

**FIGURE 1:**
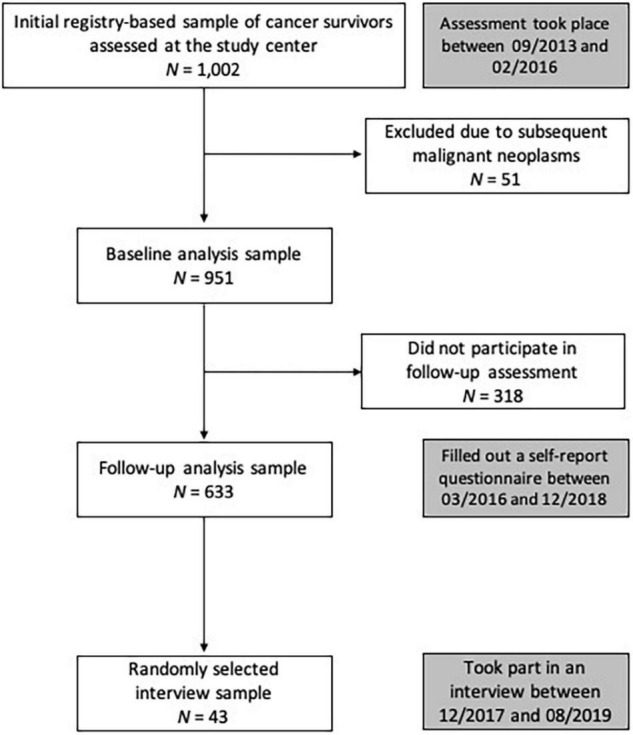
Participant flow and timeline. Between 2013/09 and 2016/02, 1,002 CCS were examined at the study center. After excluding 51 individuals due to subsequent malignant neoplasms, the baseline sample comprised 951 CCS. The current study analyzed the data collected via self-report questionnaire, which was sent to participants more than 2 years later (follow-up assessment) and completed by 633 individuals. These follow-up data constitute the core data base of the present study. We selected a subsample of 43 participants for in-depth interviews (following pre-specified categories to represent the heterogeneity of the sample, e.g., gender, cancer diagnosis, current health, participation in medical follow-up). Within these categories, interview participants were drawn randomly.

Childhood cancer survivors were recruited in cooperation with the German Childhood Cancer Registry (GCCR). The GCCR was established in 1980. German CCS were eligible for participation if diagnosed with neoplasia according to the International Classification of Childhood Cancer (ICCC-3) (Steliarova-Foucher et al., 2005) between 1980 and 1990 before the age of 15 years, and if they had received antineoplastic treatment at one of 34 participating pediatric cancer centers. Out of 2,894 eligible CCS, 1,002 accepted the study invitation. All gave written informed consent for participation and data retrieval. Participants who took part in the interviews also consented to the audio recordings and the publication of interview excerpts. Figure 1 shows the participant flow.

### Measures and Assessment

Sociodemographic information was collected *via* self-report. Cancer-related data (diagnoses, age at diagnosis) was retrieved from primary health records of former treating medical centers and/or centrally documented individual therapy data available at the Society for Pediatric Oncology and Hematology’s (GPOH) study centers. Retrieved data was validated by trained medical staff.

Participants completed a standardized 5.5-h examination including cardiovascular and clinical phenotyping (carried out by certified medical technical assistants according to standard operating procedures) to detect physical illnesses and risk factors [this procedure is described elsewhere (Faber et al., 2018)].

The *questionnaire* contained the question “Do you regularly attend medical follow-up care to detect somatic secondary diseases and risks related to your past cancer disease and its treatment?” If participants answered “yes,” they were asked to detail the involved specialists and the frequency of their visits. If they answered “no,” they were asked to elaborate (*via* free text) about the reasons.

Our *interview* approach was informed by Grounded Theory (Corbin and Strauss, 1990; Strauss and Corbin, 1998). The interviews were semi-structured and the underlying interview guide (provided as Supplementary Material) was adapted over time to refine data collection. The aim of this approach was to strike a balance between letting participants give insight into their personal situation and being able to relate the material of the interviews to the same general topics/themes. The main focus was participants’ biography in relation to their cancer diagnosis, treatment, recovery, and follow-up care. Wherever possible, we asked for example situations. All interviews were recorded and transcribed verbatim.

### Analyses

#### Quantitative Analyses

Sample characteristics are reported as absolute numbers and percentages or as means and standard deviations. Group differences were tested using χ^2^-test or analysis of variance, respectively. A multiple logistic regression model was used to examine the most relevant statistical predictors of medical follow-up attendance. Variables were chosen on the basis of previous research findings. Independent variables that were tested as potential predictors included the sociodemographic characteristics gender (coded 0 = men, 1 = women), age (in decades), time since diagnosis (in decades), level of education (based on the German Abitur which is a school-leaving diploma usually obtained after 12–13 years of school, coded 0 = lower than the German Abitur, 1 = German Abitur or higher), presence of physical illnesses (according to the medical assessments, coded 0 = not present, 1 = present), and type of primary cancer (entered as a categorical predictor; using indicator coding and defining leukemias as the reference category). We chose this multivariate approach to account for potential intercorrelations between variables of interest (e.g., of current age and physical health). Due to the small amounts of missing data (< 3% per variable), all analyses used list-wise deletion.

#### Qualitative Analyses

We used Thematic Analysis/Qualitative Content Analysis (Braun and Clarke, 2006).

*Questionnaire*: Participants’ answers were independently coded by two researchers. After familiarization with the material, we developed codes based on systematically interesting characteristics of the data. Codes were grouped together under overarching topics. We first started with the broader categories “Structural barriers” and “Emotional/motivational motives.” Based on the material, two further main categories emerged. After reviewing their content, we summarized them as “Lack of information” and “Medical follow-up was terminated by the provider.” The coders then independently assigned all statements to the now available four categories (they showed good correspondence: 87%). We tested whether the overarching topics fit without contradiction to the assigned codes and material. Conflicting ratings were resolved by discussion and a consensus coding was established. Lastly, we formulated the final titles for each thematic category, e.g., we specified “Lack of information about medical follow-up and/or its purpose.”

*Interviews*: Each transcript was coded line by line by two researchers. We coded relevant events (concerning diagnosis, treatment, stays in hospitals, appointments at doctors’ offices, follow-up visits), interactions (with medical staff and other providers such as psychotherapists), and decisions (e.g., with regard to continuing/stopping care). After the 43rd interview, we had reached the point of saturation (Glaser and Strauss, 1967) as the interview did not produce any novel information.

In order to synthesize information on individual health care trajectories, we first extracted all interview passages containing events and decisions having to do with medical care from the transcripts. Participants had not always retold their health care biography in chronological order, but we reconstructed an individual trajectory for each participant which we summarized in the form of a timeline. We then grouped them according to their similarities. The three emerging categories were “Continuously cared for,” “Return to care after discontinuation,” and “Dropped out of care.” Coders’ classifications showed almost perfect correspondence (95%) before establishing a consensus rating.

## Results

Data were available for 633 CCS (281 women, 44.4% of the sample). Their characteristics are presented in Table 1.

**TABLE 1:** Participant characteristics.

	Childhood cancer survivors (*N* = 633)
**Sociodemographic information (*n*, %)**	
Women	281 (44.4)
**Age at study enrolment**	
20–29 years	149 (23.5)
30–39 years	359 (56.7)
40–49 years	125 (19.7)
High school education	389 (61.5)
Married	233 (36.9)
**Disease-related information**	
Age at cancer diagnosis (*M*, *SD*)	6.34 (4.38)
Time since cancer diagnosis (*M*, *SD*)	28.07 (3.21)
**Diagnosis (*n*, %)**	
*Leukemias*	267 (42.2)
*Central nervous system tumors*	84 (13.3)
*Lymphomas*	64 (10.1)
*Others*	218 (34.4)
**Physical health**	
≥1 chronic health condition	90 (14.2)

*Sample characteristics are reported as absolute numbers and percentages or as means (M) and standard deviations (SD).*

### Attendance of Medical Follow-Up

Half of the participants (*n* = 314, 49.6%) reported having some kind of medical follow-up. The only statistically significant predictor of current attendance of medical follow-up was age, with higher age being positively related to attendance (Table 2).

**TABLE 2:** Results of the simultaneous logistic regression model of medical follow-up attendance.

	Dependent variable: Attendance of medical follow-up (314 observations)		
Independent variables:	OR	95% CI OR	*p*
Type of diagnosis (reference category: Leukemias)			0.95
-Central nervous system tumors	0.87	0.46; 1.69	0.62
-Lymphomas	0.93	0.61; 1.42	0.83
-Others	1.00	0.53; 1.81	0.99
Gender	1.43	0.98; 1.90	0.055
Age at examination (in decades)	1.44	1.02; 2.03	**0.040**
Time since cancer diagnosis (in decades)	0.94	0.68; 1.29	0.68
Level of education	0.73	0.50; 1.07	0.09
Presence of physical illness	1.30	0.85; 1.96	0.34

*Nagelkerke R^2^ = 0.48 (full model).*

*In total, 314 participants out of 633 reported medical follow-up care.*

*OR, odds ratio; CI, confidence interval.*

*Statistically significant predictors are printed in bold.*

### Reasons for Non-Attendance

Most non-attendees (*n* = 269, 84.3% of non-attendees) expressed reasons for non-attendance as part of the questionnaire survey. Four thematic categories were derived from their responses. In the following, we cite examples.

*Category 1*: Lack of information about medical follow-up and/or its purpose.

Most CCS who stated a reason for non-attendance reported a lack of information about medical follow-up and its purpose (*n* = 178, 66.2% of non-attendees who reported any reasons). They did not know about such opportunities or where to find them.

• “I didn’t know you could still be examined as an adult. I would like to do this, though.”

There was also the assumption that follow-up care’s purpose is to detect the recurrence of cancer.

• “My illness was 28 years ago, my risk of relapse is virtually non-existent.”

Participants reported the notion that medical follow-up was only relevant for CCS with manifest complaints.

• “I have no complaints that would require medical follow-up care.”

*Category 2*: Medical follow-up was terminated by the health care provider.

This was reported by *n* = 53 CCS (19.7% of non-attendees who reported any reasons).

• “The doctor in charge of my follow-up care informed me that such assessments were only useful in the first 10 years after the illness, so they ended when I was 17 years old.”
• “They sent me away 15 years ago, stating that I was healthy.”

*Category 3*: Structural barriers.

Some noted difficulties attending medical follow-up that were not directly related to lack of information (*n* = 21, 7.8% of non-attendees who reported any reasons).

• “I stopped attending medical follow-up because it took place in a city more than two hours away.”

*Category 4*: Emotional/motivational reasons.

A smaller number of study participants reported avoiding medical follow-up because of emotional/motivational reasons such as fear of the detection of a medical problem (*n* = 17, 6.3% of non-attendees who reported any reasons).

• “To be honest, I do not really want to know whether something’s wrong with my body.”
• “I am afraid that something (new) will be found.”

*Other information gathered from the text field: n* = 78 noted that they would like to attend medical follow-up. Also, the medical examination that was part of the study was generally very well received and CCS expressed wishes for regular comprehensive checks. Some also used the space to ask questions, for example: “After all, I’m healthy. Or am I?”

### Interview Data: Individual Health Care Trajectories

We interviewed 22 women and 21 men (age *M* = 33.67, *SD* = 6.42). Regarding their individual health care biographies, the interviews yielded information about diagnosis for *n* = 43, about initial treatment for *n* = 43, about early follow-up care *n* = 38, about longer-term follow-up care for *n* = 34, about transition of care for *n* = 11, and about resuming care for *n* = 12. In total, we processed 397 coded sequences from which we constructed participants’ individual trajectories. Reviewing and comparing them, three types emerged: “Dropped out of care” (*n* = 22, 51.2% of the interview sample), “Return to care after discontinuation” (*n* = 12, 27.9% of the interview sample), and “Continuously cared for” (*n* = 9, 20.9% of the interview sample).

*Group 1:* Dropped out of care (10 women and 12 men, age *M* = 37.09, *SD* = 6.71).

These participants reported no medical follow-up care. Their accounts yield insight into situations where care ends and where needs for care were not met:

• A participant who was diagnosed with sarcoma at the age of one and a half years recounted “a few years” of regular checks at the University Clinic where he had been treated. However, when he was 8 years old, his mother died unexpectedly. This was also when his follow-up care stopped: “We had other problems during this time. (…) I didn’t resume the appointments. As a teenager, I did not give my health much thought.”
• Another participant (diagnosed with a central nervous system tumor at the age of ten) reported: “When I was younger, my parents went to the follow-up visits with me. I do not remember being “formally” discharged. I think I went there every 2 years. Anyway, when I was 19, I moved to a city on the other side of Germany to start my studies and I did not even have a GP there.”
• Some reported difficulties with regard to receiving care, for example a female survivor of leukemia (diagnosed at the age of nine, her follow-up care ended care when she was 16 years old) said: “Since puberty I’m struggling with pain. My GP never took it seriously. I went to many doctors and at some point, I was diagnosed with fibromyalgia. (…) I also tried psychotherapy.” She reported that the psychotherapist did not consider her cancer history to be relevant. In her forties, she joined a support group for people with chronic pain. “I felt understood—for the first time ever.”

*Group 2:* Return to care after discontinuation (8 women and 4 men, age *M* = 31.74, *SD* = 4.14).

This group comprised participants who did not have continuous follow-up-care, but who now attend some form of medical follow-up.

• One woman (diagnosed with leukemia at the age of five) recounted: “As soon as it became a voluntary issue, I stopped going to the doctor. I did not think that I needed it. However, last year my company physician diagnosed enormously high blood pressure. Out of nowhere. It scared me immensely. Now, I regularly take medication and visit a cardiologist. I also have a complete blood count done regularly because it was recommended to me.”
• A male survivor of lymphoma (diagnosed at the age of 11) narrated that the pediatric clinic had discharged him at the age of 18. He then moved to a different city, but he remembers wanting to continue care: “I don’t know, after two years or so it just did not feel right not to have regular medical checkups.” So he requested his files, made copies, and brought them to his new general physician whom he had sought in the new city.

*Group 3:* Continuously cared for (4 women and 5 men, age *M* = 33.87, *SD* = 6.80).

These participants’ health care biographies are examples of good transitions and uninterrupted care.

• For instance, one participant (diagnosed with leukemia at the age of nine) reported a smooth transition from pediatric to adult care: “Until I was about 18 or 19 years old, I had my regular checks done at the clinic where I had been treated as a child. Someday my doctor said: Next time, you’re not coming back here, you’ll have to continue doing these checks somewhere else. Is there a GP you visit who is informed about your medical history? And I said sure, my family doctor is a very dedicated person and he knows me since birth. So, they told me in detail which organs should be regularly examined. They gave me this information in writing and I brought it to my family doctor. Since then, he has been doing the regular checks.”
• Another participant (diagnosed with nephroblastoma at the age of 7) reports coming back to the clinic until the age of 22. After that, his GP took over: “She examines my kidneys using ultrasound and also analyzes the concentration of proteins in my urine.”

*Group differences*: The three groups did not differ with regard to gender distribution (*p* = 0.45). However, we observed statistically significant age differences [*F*(2,42) = 3.65, *p* = 0.035].

*Post hoc* analyses using the Tukey *post hoc* criterion for significance showed that participants in Group 2, “Return to care after discontinuation” were older than participants in Group 1, “Dropped out of care” (*p* = 0.027). No other comparisons yielded statistically significant results.

## Discussion

We investigated medical follow-up within a large sample of CCS more than 25 years after diagnosis, with a particular focus on reasons for non-attendance. The proportion of CCS (roughly half) who attended medical follow-up was greater than in a comparable Swiss study (23%) (Michel et al., 2011). The Swiss sample comprised 1,075 survivors (46.7% women) with a larger range regarding time since diagnosis (6–36 years) and current age (20 to over 35 years, with a median age 26 at the time of the survey).

In multivariate regression, older age was a significant predictor of medical follow-up attendance. Previous studies had reported contrary results, mainly based on younger samples (Klosky et al., 2008). However, Oeffinger et al. (2004) noted that medical follow-up attendance diminished with time since initial diagnosis and treatment based on a large cohort study with a mean age of 27 years. The observation that more of the older survivors attended medical follow-up in our study could have different reasons. First, our interview data suggested that after having discontinued regular medical checks for a while, some CCS resumed care (e.g., due to emerging medical difficulties). Second, starting at the age of 35, German health care companies provide for medical check-ups every 3 years (including vaccination status, physical, blood, and urine examination) (Bundesministerium Für Gesundheit, 2020). This is important contextual information, but we do not know whether such offers played a role in older survivors finding their way back into the health care system. Analyses based on the smaller interview sample showed that CCS who had returned to care were slightly older than those who had dropped out of care. This age difference was statistically small, but it could indicate that one motivation for survivors to participate in follow-up care is that they encounter physical health issues as they get older.

Beyond this finding, there were no associations with disease-related or sociodemographic variables. This could indicate that within the German context, health care disparities (e.g., in accordance with the level of education) are not as pronounced as in other countries from which previous investigations derived. Another explanation could be that the present sample was more homogenous than samples investigated in other studies (as follow-up times were very long and we did reach only a fraction of the eligible target population).

According to participants’ reports, main reasons for non-attendance were a lack of information about follow-up care or its purpose. These answers indicate potentially dangerous misconceptions and gaps in their health knowledge (Bashore, 2004). Correspondingly, in a Swiss study, 13% of CCS considered follow-up care unnecessary (Lupatsch et al., 2016). Germany and Switzerland have comparable health care systems in many respects, and long-term care for cancer survivors is free of charge in both contexts. Against this background, it is understandable that CCS’ most decisive reasons for not attending care have to do with considerations of personal benefit and/or importance and that by comparison, other factors such as financial difficulties played no relevant role in their follow-up care attendance.

In addition, many providers did not seem to have specific expertise in caring for CCS, mirroring findings of previous studies (Henderson et al., 2010; Suh et al., 2014). A Canadian study found that GPs’ unfamiliarity with cancer survivors’ care needs was a barrier to medical follow-up care (Howard et al., 2018). CCS as well as health care providers need to know that late effects could occur decades after diagnosis and treatment. It is thus crucial to provide ongoing care. Less frequently reported reasons were barriers such as long distances and emotional-motivational motives. Long distances were previously highlighted (Nathan et al., 2016; Daly et al., 2019). In our study, we learned that many German CCS had last attended care at the pediatric oncology unit where they had been treated (often implicating long journeys). Thus, they should be supported in establishing new contacts at their current place of residence.

The interviews mirrored the questionnaire findings and provided a deeper understanding of them: Both the group of participants who had dropped out of care and the group of participants whose care had been interrupted had experienced the barriers to care that had already been voiced by the larger sample (Categories 1, Lack of information about medical follow-up and/or its purpose, and 2, Medical follow-up was terminated by the health care provider): There were cases in which care had been discontinued by the provider, or the provider did not ensure ongoing care, e.g., when CCS became too old for the pediatric care setting. Further, there was often a lack of information regarding the purpose of long-term follow-up care on the part of the survivor. Therefore, it was not possible for them to make an informed decision in the sense that they could have worked toward ongoing care with their doctor. Also in line with the frequency of the questionnaire responses, Categories 3, Structural barriers, and 4, Emotional/motivational reasons played a minor role in the interviews.

Thus, interview data strengthened the impression that decades after treatment and initial diagnosis, long-term CCS’ health care situation was oftentimes shaped by fortunate circumstances (e.g., by having committed/informed general practitioners or company doctors) rather than by providers following guidelines or experts’ recommendations. We had expected non-attendance to be strongly related to fears about one’s health or wishes to avoid reminders of the disease and its treatment (Zebrack et al., 2004; Rosenberg-Yunger et al., 2013). Although participants may not have voiced all of their concerns, they voluntarily underwent a medical investigation in the context of the study and many expressed to have appreciated this offer.

Our participants’ reports, both based on the questionnaire responses provided by the larger sample and the in-depth interview with a subsample, suggest that not attending medical follow-up might less often be a conscious decision than simply lack of knowledge as this was the most frequently reported reason for non-attendance. Thus, there is the potential to motivate more survivors to attend medical follow-up (for instance by GPs raising the issue, or by better cooperation among health care professionals). Along these lines, promoting the notion that medical follow-up serves prevention and the maintenance of quality of life might be helpful to encourage those who currently feel well. Additionally, if more survivors participated in some kind of follow-up, this would also help health professionals to identify survivors with mental distress and psychosocial care needs (Gianinazzi et al., 2014). This is an important issue as the disease and its treatment can have a significant impact on children’s and adolescents’ development, for example by complicating or delaying the attainment of psychosocial milestones, by affecting identity development, and by interrupting initial close relationships with peers. Studies have shown that long-term CCS have an elevated risk to experience mental distress (Burghardt et al., 2019) compared to general population samples and many of them have psychosocial difficulties (Brinkman et al., 2018) as well, e.g., social isolation (Ernst et al., 2021).

Therefore, the relevant guidelines recommend multidisciplinary follow-up care. Along these lines, future research investigating cancer survivors’ (participation in) follow-up care should also aim to yield a better understanding of the interaction of biological, social, and psychological factors in shaping their physical and mental health outcomes. For instance, they could include considerations of psychosocial development and potential risk and protective factors going beyond sociodemographic and disease-related variables, such as psychosocial development and family support (Ernst et al., 2020).

### Strengths and Limitations

The study has several limitations that need to be considered when interpreting its results. First, we excluded individuals with secondary malignancies as their treatment could have effects on physical health outcomes (the main endpoint of the CVSS study). This presents a limitation as secondary cancers are a common late effect (Oeffinger and Hudson, 2004) and were associated with follow-up care attendance (Klosky et al., 2008; Lupatsch et al., 2016). Secondly, there might be disparities influencing health care biographies that were not captured by the investigation [such as parents’ socioeconomic status and educational attainment or ethnic minority status (Barakat et al., 2012)].

We also lacked specific information about participants’ cancer (e.g., tumor location) and treatment characteristics (e.g., chemotherapeutic agents, dose/field size of radiation), making assessments of participants’ individual risk for late effects impossible. Further, our question about medical follow-up did not differentiate between providers (e.g., GP or specialist physicians) and types of care (e.g., specialized long-term follow-up care). Therefore, this study does not allow for an estimate regarding how many CCS did receive medical care in line with the current guidelines, it rather captured the bare minimum of medical follow-up care. Future research should thus assess long-term follow-up in a more detailed manner. There is also the need for more representative samples: As described, only a third of the invited CCS took part in the study and not all of them completed the questionnaire (i.e., only 22% of the eligible CCS took part in the follow-up assessment from which we drew our data). This participation rate is slightly lower than in previous surveys of German long-term CCS (Seitz et al., 2010). We cannot rule out self-selection effects limiting the generalizability of our findings to the CCS population, e.g., that healthy and well-adjusted former patients did not accept the invitation as they did not perceive it as relevant to their lives, or that CCS with ill health felt that study participation was too burdensome. It is also possible that factors related to the very questions examined in this work influenced self-selection of study participants: for example, it can be assumed that CCS who do not want to know more about their health and who are afraid of learning about potential late effects would have declined the study invitation. Also, we assume that participants knew more about medical follow-up care than other CCS (e.g., because they had already undergone an assessment at the study center when they answered the question about medical follow-up that was part of the add-on study). Thus, in this respect, the study group is not representative for German long-term CCS. Lastly, our participants had been treated before the challenges of long-term survivors came to the fore of research and practice. Today’s CCS situation might be different as international consortia have been formed to improve CCS’ care and quality of life (Hjorth et al., 2015) and Germany now also has specialized late effects clinics in some locations (Gebauer et al., 2018).

This is the first investigation of medical follow-up attendance and reasons for non-attendance in a large sample of German CCS > 25 years after diagnosis. The qualitative approach allowed for the identification of relevant topics based on CCS’ circumstances and their subjective perceptions. The results indicated great needs for more information about medical follow-up among CCS. CCS are a vulnerable group and should be educated about the management of cancer-related health risks. Given the growing numbers of CCS who reach middle and late adulthood, it is a matter of urgency to acknowledge their care needs and to offer appropriate services.

## Data Availability Statement

The datasets presented in this article are not readily available because the written informed consent of the study participants is not suitable for public access to the data and this concept was not approved by the local data protection officer and ethics committee. Access to data at the local database in accordance with the ethics vote is offered upon request at any time. Interested researchers make their requests to the Principal Investigators of the CVSS/PSYNA study (Philipp.Wild@unimedizin-mainz.de). Requests to access the datasets should be directed to PW, Philipp.Wild@unimedizin-mainz.

## Ethics Statement

The studies involving human participants were reviewed and approved by Rhineland-Palatinate Chamber of Physicians. The patients/participants provided their written informed consent to participate in this study. Written informed consent was obtained from the individual(s) for the publication of any potentially identifiable images or data included in this article.

## Author Contributions

ME and MEB: methodology. ME: formal analysis and investigation. ME, EB, and MEB: writing—original draft and preparation. PSW, JF, and HM: writing—review and editing. All authors contributed to the conceptualization of the study.

## Conflict of Interest

The authors declare that the research was conducted in the absence of any commercial or financial relationships that could be construed as a potential conflict of interest.

## Publisher’s Note

All claims expressed in this article are solely those of the authors and do not necessarily represent those of their affiliated organizations, or those of the publisher, the editors and the reviewers. Any product that may be evaluated in this article, or claim that may be made by its manufacturer, is not guaranteed or endorsed by the publisher.
