# Supplementary material for: A Mixed-Methods Investigation of Medical Follow-Up in Long-Term Childhood Cancer Survivors: What Are the Reasons for Non-Attendance?
Source: Front Psychol. 2022 Mar 14;13:846671. doi: 10.3389/fpsyg.2022.846671 (PMC8967151; doi:10.3389/fpsyg.2022.846671)
Supplement: Supplementary file 1 [file Data_Sheet_1.PDF]

SUPPLEMENTAL MATERIAL FOR "A mixed-methods investigation of medical follow-up in long-term childhood cancer survivors: What are the reasons for non-attendance?"

Mareike Ernst[1*], Elmar Brähler[1], Jörg Faber[2], Philipp S. Wild[3, 4, 5], Hiltrud Merzenich[6], Manfred E. Beutel[1]

[1]University Medical Center of the Johannes Gutenberg-University Mainz, Department of Psychosomatic Medicine and Psychotherapy, Mainz, Germany
[2]University Medical Center of the Johannes Gutenberg-University Mainz, Department of Pediatric Hematology/Oncology/Hemostaseology, Center for Pediatric and Adolescent Medicine, Mainz, Germany
[3]University Medical Center of the Johannes Gutenberg-University Mainz, Preventive Cardiology and Preventive Medicine-Department of Cardiology, Mainz, Germany
[4]University Medical Center of the Johannes Gutenberg-University Mainz, Center for Thrombosis and Hemostasis, Mainz, Germany
[5]DZHK (German Center for Cardiovascular Research), partner site Rhine-Main, Mainz, Germany
[6]University Medical Center of the Johannes Gutenberg-University Mainz, Institute for Medical Biostatistics, Epidemiology and Informatics, Mainz, Germany

*** Correspondence:**
Dr. Mareike Ernst
Mareike.Ernst@unimedizin-mainz.de
ORCID 0000-0003-4952-9717
Department of Psychosomatic Medicine and Psychotherapy, University Medical Center of the Johannes Gutenberg-University Mainz, Untere Zahlbacher Str. 8, 55131 Mainz, Germany
Telephone: +49 6131 17-7649

English translation of the semi-structured interview guide used in the PSYNA study[1]

Notes for the interviewer:

- *Preparation*: Read the free text field of the questionnaire and check when it was filled out, note potential topics for exploration
- *During the interview:* Practice active listening and strive for depth of information, not only breadth, by asking participants to elaborate where possible (e.g., What comes to mind when you think back to this situation? Could you describe an exemplary situation?)

1. **Opening**
    - Introduction
    - Thanks for participation
    - Information about recording, consent to record
    - Timeframe: up to 45 min

You can end the interview at any time without explanation. If there are any topics you do not want to talk about or questions you do not want to answer, this is completely understandable. Please just let me know.

I am interested in your personal experiences, there are no "right" or "wrong" answers. What *you* experienced and how you personally dealt with it is what it's all about.

[1] Notes for readers: Information about the risk of late effects had been provided at the beginning of the study, together with the study invitation. All childhood cancer survivors who took part in the study then underwent an extensive health check at the study center during which all medical examinations and their findings were explained. At a later measurement point, they filled out a questionnaire assessing mental health, psychosocial aspects, and participation in follow-up care (participant flow and timeline are shown in Fig. 1 in the manuscript). Questionnaire information was available to the interviewer. The questions included in this interview guide were not necessarily asked in the presented order; further, not all questions could be applied to all participants.

There are several topics I would like to address: First, your experiences dealing with the disease (the diagnosis process, primary treatment, and beyond), and secondly, your follow-up care experiences or needs and how you fared throughout the process. Thirdly, I would like to learn more about your current situation and your care needs.

2. Diagnosis and initial treatment experiences

- How old were you when you were diagnosed?
- Do you remember how you received the diagnosis?
- How long was your treatment?
- What do you remember of the treatment you received/the time in which you were treated?

If you look back, from today's perspective:

- What was the most formative experience during that time?
- What helped you the most during that time? (E.g., family, friends, primary care physician, psychotherapist, ...)

- Is there something you were missing, medically or psychologically? / Can you think of something you would have needed in addition?
- Were there times when you would have needed medical or psychological support? / Can you remember times that were difficult for you (emotionally)?

3. Follow-up care experiences

Now I would like to talk about the time after the acute treatment. It is common for many cancer survivors to go for check-ups, at first more and after some time less frequently. In this

first time, the focus is on potential cancer recurrence. Later, the monitoring/early detection of potential late effects become more important

3.1 Longer ago

Gathering of *"hard facts"*:

- Do you remember the transition from acute treatment to follow-up care?
- Can you still remember follow-up care appointments?
- How did they go? / How did you experience them?
- Who was with you?

- Which follow-up care appointments did you find most helpful? Which ones were less helpful?
- What were your most important experiences in follow-up care (positive and negative experiences)?

Exploration of *emotions* and *cognitions* in/about follow-up care:

- If you think back, how did you feel / how was it for you to go to these appointments?
- (If applicable:) Could you please tell me about the day before and the day of the follow-up and the day of the follow-up itself? (What do you remember? / How did you feel? / What thoughts did you have?)
  (perhaps suggest: for some, it is stressful, provokes anxiety/reminds them of hard times in their lives, for others, it signifies a milestone of survivorship - how did you experience it?)
- Did you feel like you were being well taken care of?
- What would have made this experience better? Was there anything you felt was missing?

3.2 Current

For survivors in care:

- Could you describe to me what your follow-up care looks like at the moment?
- In which ways did it change over time? (E.g., frequency, specialist doctors, locations, perhaps also their feelings/thoughts about it…)

- When was the last time you went?
- How satisfied are you with the current follow-up care you are receiving? (Professionally and on a relationship level)

For survivors not in care:

- How old were you *when* it ended / how long ago was that?
- Could you describe to me *how* it ended?
- *Why* did it end? (Examples: Was it a conscious decision? / Who made this decision?)
  - If own decision/stopped on their own:
    - Could you explain your decision process? / What factors contributed to this decision? (E.g., importance of follow-up, burden of participation, e.g., because of travel)
  - If from the doctor's side:
    - What reasons were communicated to you?
    - Did they recommend to continue any kind of care elsewhere?
    - Were you given any further advice? (e.g., with regard to health care, health behavior, etc.)

For all:

- Do you currently feel that you are well taken care of?
- Are there any services you are missing?

4. Exploration of current subjective health status and concerns

- What information do you have about potential late effects of disease and treatment?
- What is the source of this information? (If applicable: Has there been an assessment/communication of your individual risk/risk factors?)
- Do you talk about these topics with your doctors? (If applicable, depending on previous answers: primary care physician, specialist doctors, …)

- How would you rate your overall health (on a scale from 0 to 10, 10 indicating best possible health status)?

- Did you ever experience any late effects / any kinds of symptoms or illnesses that you/your doctors conceive of as late effects? Do you experience them currently?
- Are you concerned about possible late effects?

(If participants do not mention the materials included in the study invitation of their own account:)

It has been a while now, but as we first invited you to take part in this study, you also received information about potential late effects.

- What was it like for you to receive this information?
- Was this the first time you heard about late effects?

As a part of this study, you also took part in an extensive medical examination at the study center. We thank you very much for that.

- Do you remember what informed your decision to take part in this assessment?
- How did you feel before this appointment? / How did you feel during it?
- Is there any important piece of information you received on this day?

- Did the results / did study participation change anything about how you think about your health / your status as a long-term survivor of childhood cancer? (E.g., did they inform any changes in health behavior, lifestyle, or follow-up care?)

4. Conclusion of the interview

Summarize the main points, ask whether participant agrees with the summary

- Did I forget anything that is important to you? / Is there anything you would like to add?
- What would you say were barriers to care or gaps in care for you personally?

- How do you feel now, after this conversation?

(If the conversation has upset the participant: We can offer psychological support from an experienced therapist. Ask specifically: Would you like to speak to someone now? / Would you like to make an appointment at another time? If not: You can also call this number back should you change your mind.)

Closing:

Thank you so much for your time. If you have any further questions or comments, please do not hesitate to reach out again (by email or by phone).

Have a great day.
